# Anisotropy in the thermal hysteresis of resistivity and charge density wave nature of single crystal SrFeO_3-δ_: X-ray absorption and photoemission studies

**DOI:** 10.1038/s41598-017-00247-z

**Published:** 2017-03-13

**Authors:** S. H. Hsieh, R. S. Solanki, Y. F. Wang, Y. C. Shao, S. H. Lee, C. H. Yao, C. H. Du, H. T. Wang, J. W. Chiou, Y. Y. Chin, H. M. Tsai, J.-L. Chen, C. W. Pao, C.-M. Cheng, W.-C. Chen, H. J. Lin, J. F. Lee, F. C. Chou, W. F. Pong

**Affiliations:** 1 Department of Physics, Tamkang University, Tamsui, 251 Taiwan; 2 Department of Physics, National Tsinghua University, Hsinchu, 300 Taiwan; 3 Department of Applied Physics, National University of Kaohsiung, Kaohsiung, 811 Taiwan; 4 National Synchrotron Radiation Research Center, Hsinchu, 300 Taiwan; 5 Center for Condensed Matter Sciences, National Taiwan University, Taipei, 106 Taiwan; 6 Centre of Material Sciences, Institute of Interdisciplinary Studies, University of Allahabad, Allahabad, 211002 Uttar Pradesh India

## Abstract

The local electronic and atomic structures of the high-quality single crystal of SrFeO_3-δ_ (δ~0.19) were studied using temperature-dependent x-ray absorption and valence-band photoemission spectroscopy (VB-PES) to investigate the origin of anisotropic resistivity in the ***ab***-plane and along the ***c***-axis close to the region of thermal hysteresis (near temperature for susceptibility maximum, T_m_~78 K). All experiments herein were conducted during warming and cooling processes. The Fe *L*_3,2_-edge X-ray linear dichroism results show that during cooling from room temperature to below the transition temperature, the unoccupied Fe 3*d e*_g_ states remain in persistently out-of-plane 3*d*_3z_^2^_-r_^2^ orbitals. In contrast, in the warming process below the transition temperature, they change from 3*d*_3z_^2^_-r_^2^ to in-plane 3*d*_x_^2^_-y_^2^ orbitals. The nearest-neighbor (NN) Fe-O bond lengths also exhibit anisotropic behavior in the ***ab***-plane and along the ***c***-axis below T_m_. The anisotropic NN Fe-O bond lengths and Debye-Waller factors stabilize the in-plane Fe 3*d*_x_^2^_-y_^2^ and out-of-plane 3*d*_3z_^2^_-r_^2^ orbitals during warming and cooling, respectively. Additionally, a VB-PES study further confirms that a relative band gap opens at low temperature in both the ***ab***-plane and along the ***c***-axis, providing the clear evidence of the charge-density-wave nature of SrFeO_3-δ_ (δ~0.19) single crystal.

## Introduction

Recently, SrFeO_3-δ_ based colossal magnetoresistance (CMR) materials have attracted much interest owing to their potential applications in the next generation of magnetic data storage read heads, and because of the research goal of elucidating the microscopic origin of CMR^1–4^. Extensive research on these CMR materials and associated electron structures that has been performed for the last decade is still ongoing^5–10^.

The structural, magnetic and transport properties of SrFeO_3-δ_ vary dramatically with oxygen content and the valence state of Fe^7, 11–13^. The known phases of SrFeO_3-δ_ include stoichiometric SrFeO_3_ (δ = 0), which has a cubic perovskite structure with a valence state of Fe^4+^, and oxygen-deficient phases with tetragonal (δ = 0.125), orthorhombic (δ = 0.25) and brownmillerite-type (δ = 0.50) crystal structures^1, 2^. Certain amount of oxygen result in the coexistence of these phases. The tetragonal phase comprises FeO_6_ octahedra, distorted/tilted FeO_6_ octahedra and square pyramidal FeO_5_^2^. The orthorhombic phase reportedly includes distorted FeO_6_ octahedra and square pyramidal FeO_5_, in which Fe has valence of 3+ and 4+, respectively. The brownmillerite-type phase has tetrahedrally and octahedrally coordinated Fe sites with an Fe valence state of 3+^2^. In these oxygen-deficient SrFeO_3-δ_ systems, giant negative magnetoresistance has been observed in the tetragonal phase around a coincident charge-spin ordering transition temperature^1, 2^. This transition exhibits thermal hysteresis of resistivity and magnetization, suggesting a first-order phase transition. The thermal hysteresis around the transition temperature (close to the temperature for susceptibility maximum, T_m_) has been attributed to the coexistence of antiferromagnetic and paramagnetic phases^5, 6^. Further, a change in the lattice structure that originates from the charge ordering (CO) transition has been observed in Raman, far-infrared ellipsometric and neutron diffraction studies^1, 2, 14^. In transition metal oxides, CO-associated lattice distortion, which depends on the electron-lattice coupling strength, can cause orbital ordering (OO) so CO has been found to coexist with OO^15–18^. Bao *et al*. presented evidence of a CO-induced structural phase transition in single crystalline (Bi,Ca)MnO_3_^15^. Chi *et al*. also observed strong enhancement of orthorhombic distortion owing by CO, which allows OO to be established in single crystals of Pr_0.5_Ca_1.5_MnO_4_^17^. Similar observations have been made concerning other CO transitions, specifically, the charge-disproportionation of Fe ion and/or the formation of a charge-density-wave (CDW) in La_1−x_Sr_x_FeO_3_^3, 19^. However, in (La_2−2x_Sr_1+2x_)Mn_2_O_7_, competing local lattice distortion and the hopping of charge carriers generate comparable amounts of Mn out-of-plane 3*d*_3z_^2^_-r_^2^ and in-plane 3*d*_x_^2^_-y_^2^ orbitals in the metallic phase^16^. Therefore, the electrical and magnetic properties of (La_2−2x_Sr_1+2x_)Mn_2_O_7_ depend on not only the out-of-plane but also the in-plane Mn 3*d* states. Clearly, these orbital effects influence the electronic properties of the sample, resulting in peculiar behavior that is associated with the resistivity-related phenomena^20–22^. Chen *et al*. used a theoretical model of iron pnictides to show that unequal *d*_xz_ and *d*_yz_ orbital populations can result in anisotropic resistivity^23^. They further noted that such preferable orbital states can be identified by x-ray linear dichroism (XLD)^23^. However, the complex interplay between the mechanisms that drive OO and cause these orbital effects remains unresolved^24–26^. As discussed above, the tetragonal phase of SrFeO_3-δ_ undergoes a first-order spin-charge ordering transition that is accompanied by a change in lattice structure, which exhibits exotic electrical and magnetic properties^1^. Most spectroscopic investigations of SrFeO_3-δ_-based materials have involved varying the oxygen content and substitution at Sr or Fe sites; very few experimental and theoretical studies that involve the metal-to-insulator (MI)/semiconductor transition in SrFeO_3-δ_^3, 4, 19^. Using resonant x-ray scattering (RXS), Martin *et al*. elucidated the CDW nature of the CO transition (MI/semiconductor transition) in La-substituted SrFeO_3_^19^. However, their studies did not provide evidence of band gap opening in the formation of CDW^19^.

In this context, an investigation of the local electronic and atomic structures of tetragonal SrFeO_3-δ_ by x-ray polarization (***E***)-dependent absorption and valence-band photoemission spectroscopy (VB-PES), can provide insight into the preferred orbital of Fe 3*d* states, lattice distortion that is related to the anisotropy of resistivity in the thermal hysteresis region, and band gap opening as a function of temperature. Therefore, a detailed study of single crystalline SrFeO_3-δ_ (δ~0.19) is conducted herein using x-ray diffraction, magnetization, resistivity, x-ray absorption near-edge spectroscopy (XANES), XLD, extended x-ray absorption fine structure (EXAFS) and VB-PES. All experiments were carried out during warming and cooling runs with normal incidence (***E***//***ab***-plane, *θ* = 0°) and incidence at a glancing angle (near ***E***//***c***-axis, *θ* = 70°).

## Results and Discussion

**Figure 1:**
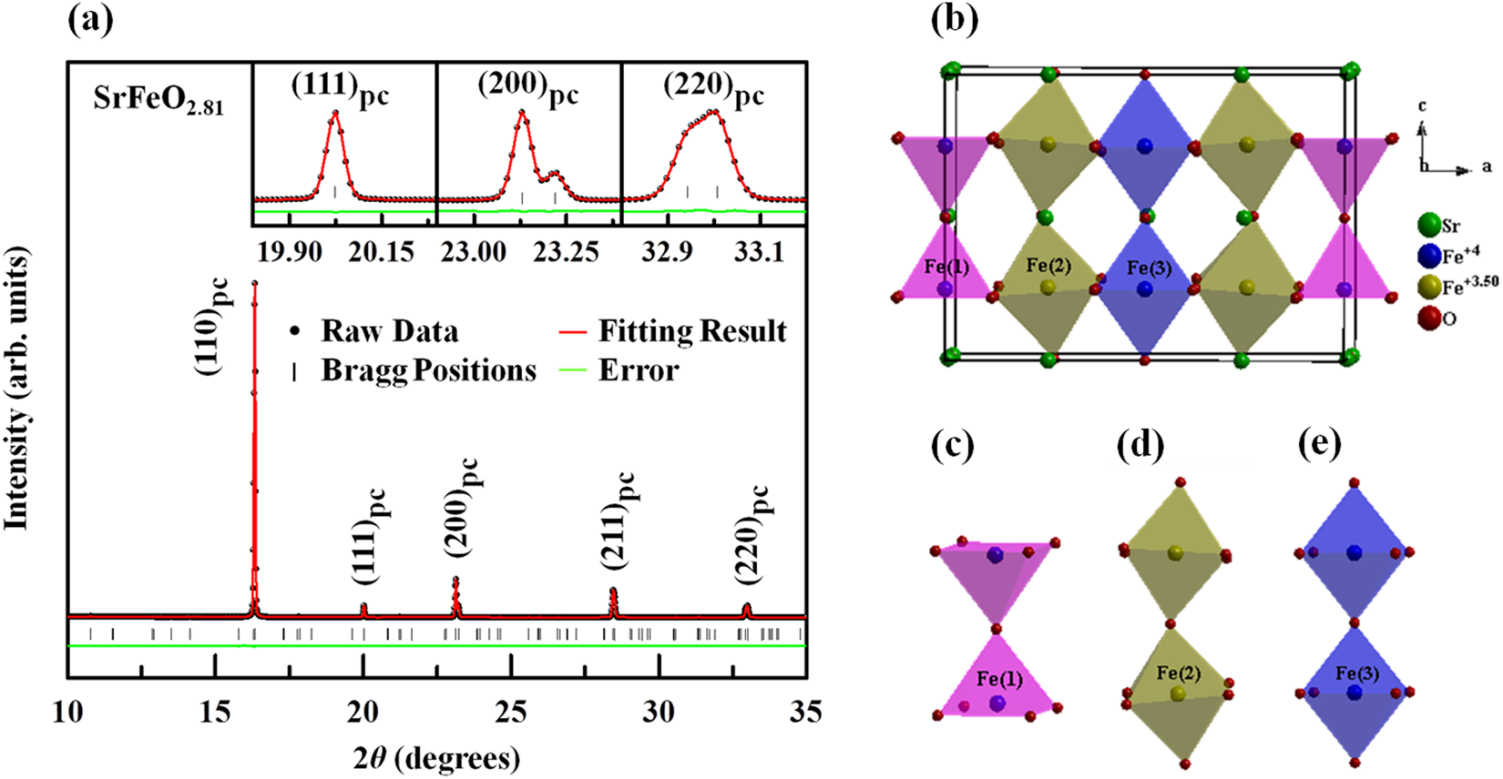
(**a**) Observed (red dots), calculated (black line) and difference (bottom green line) patterns of SrFeO_2.81_, obtained using Lebail refinement of synchrotron X-ray powder diffraction data. Vertical check marks above difference profiles indicate Bragg reflections. Insets magnify selected pseudocubic reflections, (**b**) crystalline structure of SrFeO_2.81_; valence Fe^4+^ is attributed to Fe(1), Fe(3) and Fe^3.5+^ is attributed to Fe(2), based on previous work^1^, (**c**) FeO_5_ square pyramidal, (**d**) FeO_6_ distorted octahedra, and (**e**) FeO_6_ octahedra.

Figure 1(a) presents Lebail fits^27–29^ of the X-ray powder diffraction (XRPD) of SrFeO_3-δ_ sample at room temperature based on the tetragonal *I*4/*mmm* space group model^12^. The refined lattice parameters are ***a*** = ***b*** = 10.9320(5) Å and ***c*** = 7.7012(5) Å. The insets in Fig. 1(a) magnify some of the Bragg reflections. The singlet nature of the (111)_pc_ peak and the doublet nature of the (200)_pc_ and (220)_pc_ (pc = pseudocubic) peaks are characteristic of the tetragonal phase. Figure 1(b) schematically depicts the crystal structure of the tetragonal SrFeO_3-δ_, which includes Fe sites of three types pyramidal, [Fe(1), Fig. 1(c)], distorted/tilted octahedral, [Fe(2), Fig. 1(d)] and octahedral [Fe(3), Fig. 1(e)] with valence states of Fe^4+^, Fe^3.5+^and Fe^4+^, respectively on the basis of the Mössbauer spectroscopy results that were obtained by Lebon *et al*.^1^ and the bond valence calculations that were made by Hodges *et al*.^12^.

**Figure 2:**
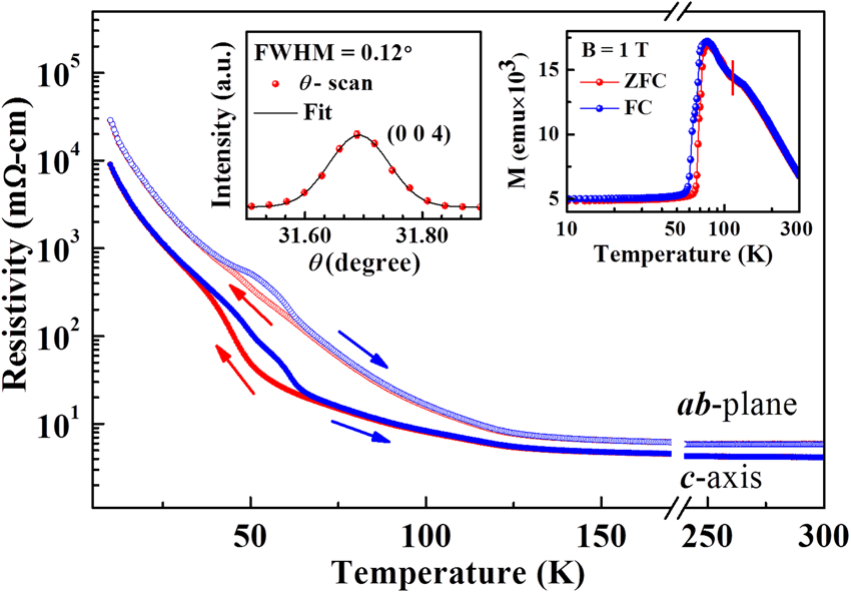
Temperature-dependence of resistivity of a single crystal of SrFeO_2.81_, measured in ***ab***-plane and along ***c***-axis. Top inset shows temperature-dependence of magnetic susceptibility (χ) measured along ***c***-axis in ZFC and FC runs in a magnetic field of 1 Tesla, and bottom inset presents room-temperature x-ray diffraction profile showing (004) Bragg peak obtained in *θ-*scan.

Figure 2 plots the temperature-dependence of resistivity in the ***ab***-plane and along the ***c***-axis. The top right-inset plots the magnetic susceptibility (χ) of the crystal, which was also measured along the ***c***-axis in a magnetic field of 1 Tesla in the zero-field-cooled (ZFC) and field-cooled (FC) runs. The top left-inset shows the room-temperature x-ray diffraction profile of the (004) Bragg peak of a single crystal of SrFeO_3-δ_. The sharpness of the peak (full width at half maximum~0.12°) indicates the high-quality of the single crystal^27^. Resistivity and magnetization measurements presented in Fig. 2 are consistent with those reported by Lebon *et al*. for SrFeO_3-δ_ (δ = 0.19) crystal and therefore reveals that the oxygen content of the single crystal sample is close to that of SrFeO_2.81_^1, 2^. A sharp rise in the susceptibility close to the temperature for the susceptibility maximum (T_m_~78 K, top right-inset) indicates a transition from the paramagnetic phase to the antiferromagnetic phase and is consistent with earlier studies^1, 2, 8^. The ZFC and FC curves exhibit thermal hysteresis, revealing the first-order nature of the phase transition. Thermal hysteresis (thermal hysteresis region, ΔT~20 K) in the temperature-dependence of susceptibility in SrFeO_2.81_ has been attributed to the coexistence of paramagnetic and antiferromagnetic phases^5, 6^. Additionally, a peak that is characteristic of the residual cubic phase has also been observed at 130 K (indicated by the bar herein) in the temperature-dependence of χ^1, 2, 8^. However, RXS has recently been performed by co-authors of this work, and their results suggested that the transition at ~130 K is related to the weak localization of the charge on Fe^3.5+^, which is also magnetically active and leads to an anomaly in the paramagnetic region of susceptibility^13^. It is evident from Fig. 2 that during the warming and cooling processes resistivity exhibits thermal hysteresis both in the ***ab***-plane and along the ***c***-axis. At room temperature (paramagnetic phase) and ~10 K (antiferromagnetic phase), SrFeO_2.81_ has a resistivity of ~4 and ~10^4^ mΩ-cm, respectively, reflecting typical metallic and semiconducting behaviors. According to the crystal structure in Fig. 1(b), at room temperature, SrFeO_2.81_ has three Fe sites with two valence states Fe^4+^ and Fe^3.5+^. The low resistivity in the paramagnetic phase is attributed to the delocalized electronic system with a high electron density of the Fe^3.5+^ state and the low electron density in the Fe^4+^ state, as determined by Mössbauer spectroscopy and refined room temperature neutron powder diffraction^1, 2, 12^. Moreover, the resistivity of the SrFeO_2.81_ crystal, which is independent of the warming or cooling of the sample and the measurement path, begins to increase as the temperature falls below ~130 K and is consistent with the anomaly in the magnetization data in Fig. 2. The change in resistivity as the temperature declines is reportedly associated with the beginning of the CO transition and similar to that associated with a MI phase transition^13^. Another signature of the phase transition is observed below ~78 K. This transition is accompanied by a thermal hysteresis that is characteristic of a first-order phase transition. According to the phenomenological theory of phase transition across a first-order MI phase transition, as the temperature is reduced, the volume fraction of the insulating phase increases but the metallic phase does not disappear (According to the results of Mössbauer spectroscopy, the calculated fraction of Fe^3.5+^ in the paramagnetic/metallic phase is ~55%, decreasing to ~10% in the antiferromagnetic/insulating phase. Lebon *et al*.^1^ found that the residual ~10% is caused by an oxygen deficiency), which results in the coexistence of two electronic phases over a certain temperature range, giving rise to the hysteresis of electronic properties^30, 31^. A recent study by Lee *et al*.^13^ on SrFeO_2.81_ with majority tetragonal phase revealed that magnetic and charge-related degrees of freedom are coupled with each other and thermal hysteresis in the resistivity is associated with the commensurate-to-incommensurate CO transition (The delocalized Fe^3.5+^ state with fractional valence changes to localized Fe^3+^ and Fe^4+^ states upon the CO transition). Therefore, the paramagnetic-to-antiferromagnetic transition in SrFeO_2.81_ coincides with a commensurate-to-incommensurate CO transition^13^. As expected, the resistivity of a single crystal of SrFeO_2.81_, plotted in Fig. 2, in the ***ab***-plane is always larger than that along the ***c***-axis, because the ***a*** and ***b*** lattice parameters, as reported by Reehuis *et al*.^14^, are greater than ***c*** at all temperatures, possibly lessening the conductivity of electrons. However, the results herein reveal another interesting phenomenon, which is a large difference between the thermal hysteresis region in the ***ab***-plane (thermal hysteresis region, ΔT~19 K) and that along the ***c***-axis (ΔT~30 K). The anisotropy or direction-dependence of resistivity in the thermal hysteresis region of a single crystal of SrFeO_2.81_ is not mostly explained by the coexistence of antiferromagnetic and paramagnetic phases, by the incommensurate-to-commensurate CO transition^5, 6, 13^, or in terms of the charge-disproportionation or CDW state^3, 19^. Several theoretical and experimental studies of single crystals and thin films of various materials have established that the anisotropy of resistivity is associated with OO or with the preferential occupation of orbitals, is primarily controlled by doping or lattice distortion/strains^21, 23, 26, 29–33^. Accordingly, the anisotropy of resistivity both in the ***ab***-plane and along the ***c***-axis in SrFeO_2.81_ crystal in the thermal hysteresis region is closely related to the changes in electronic and lattice structures.

**Figure 3:**
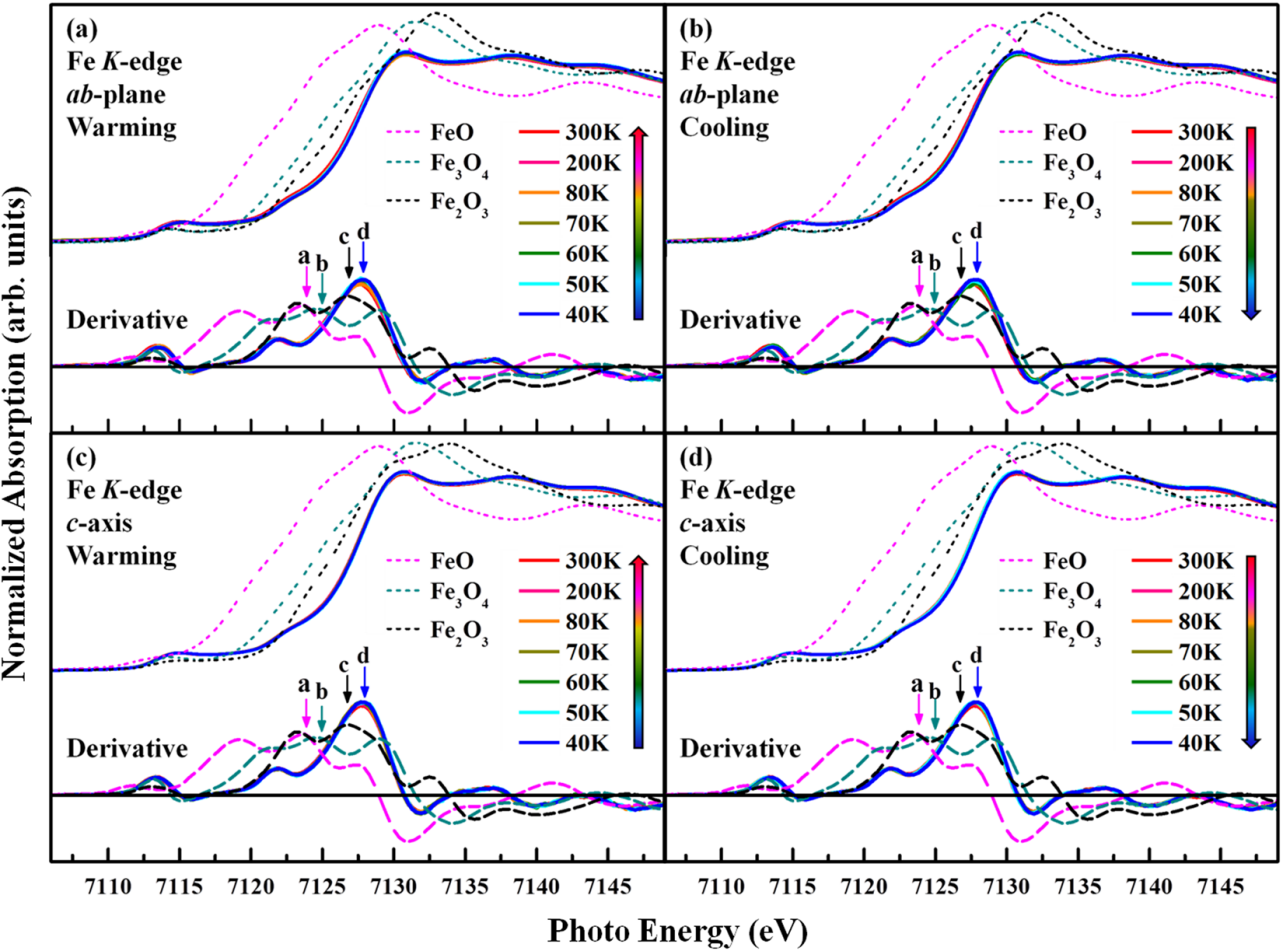
(**a–d**) The temperature-dependent Fe *K*-edge XANES spectra of single crystal SrFeO_2.81_ measured at two different angles of incidence *θ* = 0° (with electric field ***E*** parallel to the ***ab***-plane) and 70° (with electric field ***E*** nearly parallel to the ***c***-axis) on warming and cooling process. Corresponding spectra were obtained for FeO, Fe_3_O_4_, and Fe_2_O_3_ powder samples at room temperature with angle *θ* = 0° for reference.

Figure 3(a–d) present the temperature-dependent Fe *K*-edge XANES spectra of single crystalline SrFeO_2.81_, at two angles of incidence, *θ* = 0° and 70°, to the normal of the ***ab***-plane during warming and cooling processes. Corresponding spectra of FeO, Fe_3_O_4_, and Fe_2_O_3_ powder samples were obtained at room temperature, *θ* = 0°, for reference. According to the dipole-transition selection law, these Fe *K*-edge XANES spectra are primarily associated with the Fe 1 *s* → 4*p* transition, and the intensity of the main feature is attributable to the density of the unoccupied Fe 4*p* states. The bottom panels in Fig. 3(a–d) present the derivative results, to elucidate the variation of the valence state of the Fe ion in SrFeO_2.81_ with temperature, based on the position of the threshold feature^33^. The energy threshold of the Fe *K*-edge feature of SrFeO_2.81_ (feature **d**) is at 7127.5 ± 0.3 eV for both *θ* = 0° and 70° in warming and cooling at all temperatures; this threshold is above those of FeO/Fe^2+^ (feature **a**), Fe_3_O_4_/Fe^(8/3)+^ (feature **b**), and Fe_2_O_3_/Fe^3+^ (feature **c**). From the maximum of the derivative of the XANES spectrum, Blasco *et al*.^3^ obtained an average valence state of +4 for Fe ions in the powder sample of SrFeO_2.96_ with an energy threshold at 7127.5 ± 0.2 eV, which is close to that of SrFeO_2.81_ as displayed in Fig. 3. However, in the absence of standard Fe *K-*edge XANES for the 4+ valence state, the average valence state of Fe in SrFeO_2.81_ is determined to be between Fe^3+^ and Fe^4+^ and close to Fe^4+^. Additionally, the observed average valence state of Fe in SrFeO_2.81_, as stated above, obtained on the basis of Mössbauer spectroscopic results and the bond valence calculations, is determined to be Fe^4+^, Fe^3.5+^ and Fe^4+^ at the three crystallographic sites, in the ratio 1:2:1^1, 2, 12^. Importantly, the MI/CO transition of SrFeO_2.83±0.01_ and La_1/3_Sr_2/3_FeO_3_ has been attributed to charge-disproportionation of Fe^3.5+^ and Fe^3.66+^, respectively, and new models, based on a CDW^19^, have been proposed to explain the charge modulation in these compounds^13, 19^. However, as shown in Fig. 3(a–d), the energy of Fe *K*-edge and the line-shape of SrFeO_2.81_ do not vary much with temperature during warming or cooling. Clearly, the Fe *K*-edge XANES studies do not support the claim that charge disproportionation influences resistivity (or the MI/metal-to-semiconductor transition) below the transition temperature of SrFeO_2.81_.

**Figure 4:**
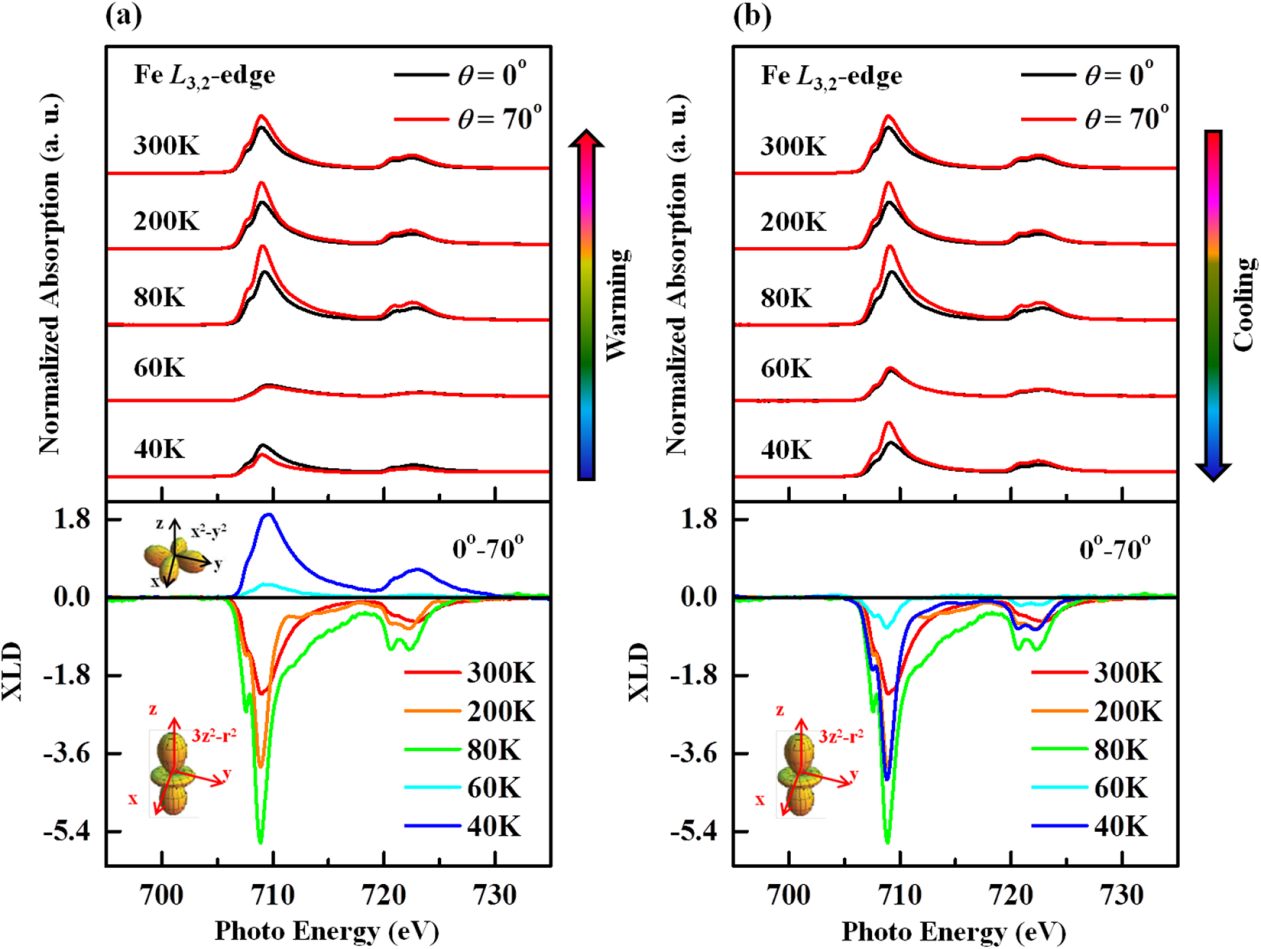
(**a**) and (**b**) Temperature-dependence of normalized Fe *L*_3,2_-edge XANES spectra of single crystal of SrFeO_2.81_ at two angles of incidence *θ* = 0° and 70° during warming and cooling. Bottom panels show corresponding XLD spectra.

**Figure 5:**
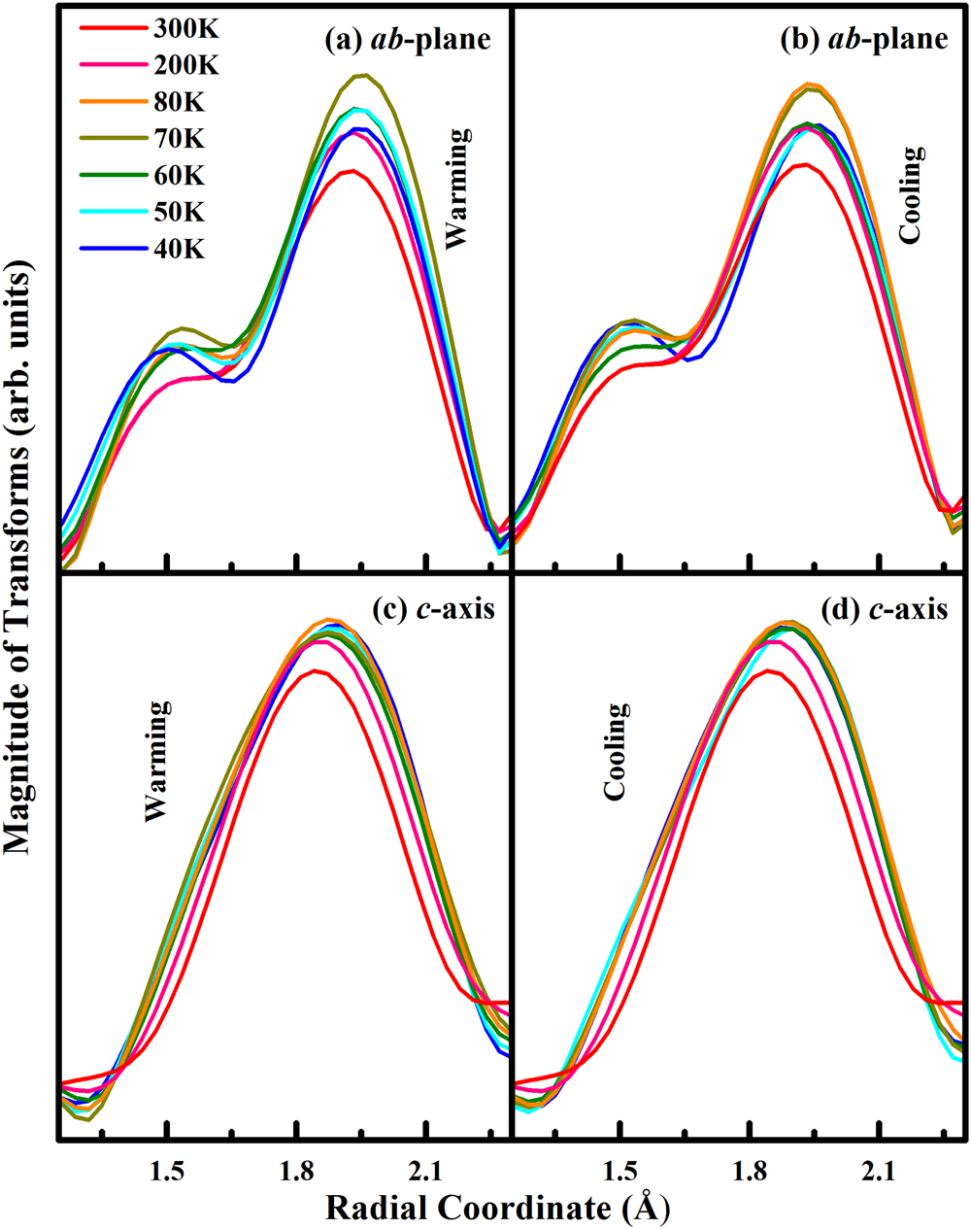
Temperature-dependence of the main FT feature **A** (corresponding to the NN Fe-O bond distance) of Fe *K*-edge EXAFS for (**a,b**) ***E***//***ab***-plane and (**c,d**) ***E***//***c***-axis in the warming and cooling process.

Figure 4(a) and (b) present the temperature-dependent Fe *L*_3,2_-edge XANES and XLD (bottom) spectra with ***E*** parallel to the ***ab***-plane (angle of incidence, *θ* = 0°) and the ***c***-axis (angle of incidence, *θ* = 70°), respectively. The Fe *L*_3,2_-edge XANES spectra include two features- an *L*_3_-edge around 708 eV and an *L*_2_-edge around 720 eV, that are separated by spin-orbital splitting. These spectra are primarily associated with the Fe 2*p* → 3*d* transition, and the intensity of the main feature is attributable to the density of the unoccupied Fe 3*d*-O 2*p* hybridized states. Notably, as displayed in Fig. 4(a) and (b), the intensity of the Fe *L*_3,2_-edge XANES spectra is greatly suppressed when ***E*** is parallel to the ***ab***-plane and almost parallel to the ***c***-axis at 60 K during warming and cooling. The Mn^3+^ ion (*d* ^4^) in an octahedral oxygen environment is well known to exhibit a large static Jahn-Teller (JT) distortion, which has an important role in the CO and MI transition^20, 21, 34^. SrFeO_2.81_ has two valence states Fe^3.5+^ and Fe^4+^, of which Fe^4+^ (*d* ^4^) is isoelectronic with Mn^3+^ and is therefore expected to exhibit a JT effect^34, 35^. Bocquet *et al*.^36^ noted that *d* ^4^ is a high-spin state with three electrons’ filling: the *t*_2g_ orbital and the remaining *e*_g_ electron itinerant, so Fe^4+^ may induce a strong JT distortion and further split the 3*d e*_g_ state into 3*d*_x_^2^_-y_^2^ and 3*d*_3z_^2^_-r_^2^ ^35, 36^. Figure S1 in the Supplementary Information schematically depicts the experiment to elucidate the in-plane and out-of-plane Fe 3*d*-O 2*p* hybridization states. The hybridizations of Fe 3*d*_x_^2^_-y_^2^-O 2*p*_x,y_ (in-plane, *θ* = 0°) and Fe 3*d*_3z_^2^_-r_^2^-O 2*p*_z_ (out-of-plane, *θ* = 70°) are probed with the electric field ***E*** parallel to the ***ab***-plane and almost parallel to the ***c***-axis, respectively. The XLD spectra (difference in XANES intensity between *θ* = 0° and *θ* = 70°) of the Fe *L*_3,2_-edge provide information on the preferred orbital of the Fe 3*d* electron, which is determined by the anisotropic effect with temperatures. The bottom panel in Fig. 4(b) reveals that the sign of the XLD feature is negative during all cooling, suggesting that Fe *e*_g_ electrons preferentially occupy the out-of-plane 3*d*_3z_^2^_-r_^2^ orbitals. However, in the warming process, the signs of the XLD spectra are reversed, being positive at 40 and 60 K, suggesting that the Fe *e*_g_ electrons preferentially occupied the in-plane 3*d*_x_^2^_-y_^2^ orbitals in the thermal hysteresis. This behavior with respect to the preferential orbital can give rise to the anisotropy that is observed in the resistivity in the thermal hysteresis region, owing to the strong coupling between the lattice distortion and the electronic structures^15–18^. Several researchers have provided evidence of structural distortions of transition-metal oxides and their correlation with OO and CO^15–18, 37, 38^. The distortion of the unit cell is primarily responsible for the lowering of the energy of either out-of-plane *e*_g_ orbitals or in-plane *e*_g_ orbitals^15–18^. In single crystals of Pr_0.5_Ca_1.5_MnO_4_, an increase in orthorhombic distortion causes OO and changes the electronic properties^17^. However, in (La_2−2x_Sr_1+2x_)Mn_2_O_7_, competition between local lattice distortion and the hopping of charge carriers is responsible for the comparable amounts of 3*d*_3z_^2^_-r_^2^ and 3*d*_x_^2^_-y_^2^ orbitals^16^. Raman, far-infrared ellipsometric and neutron diffraction studies of tetragonal SrFeO_3-δ_ have revealed a change in lattice structure as the temperature falls below T_m_^1, 2, 14^. Local lattice distortion in the SrFeO_2.81_ below T_m_ is evident in the Fe *K*-edge EXAFS results. Tetragonal SrFeO_3-δ_ has three Fe sites, in pyramidal, distorted/tilted octahedral and octahedral networks of oxygen around Fe ions. The distortion of the oxygen octahedral/pyramidal networks by a change in the nearest-neighbor (NN) Fe-O bond length and the hybridization of Fe 3*d*-O 2*p* can stabilize different out-of-plane and in-plane Fe 3*d e*_g_ orbitals, as evidenced by the temperature-dependent XLD spectra of SrFeO_2.81_ in Fig. 4^15–18^. Figure S2(a–d) in the Supplementary Information plot the temperature-dependent Fourier transform (FT) Fe *K*-edge EXAFS spectra of SrFeO_2.81_ at *θ* = 0° (***E***//***ab***-plane) and *θ* = 70° (***E***//***c***-axis). The insets present corresponding EXAFS *k* ^2^χ data. The Fe *K*-edge EXAFS spectra include main FT features (**A**, **B** and **C**) that correspond to the NN bond lengths of Fe-O, Fe-Sr and Fe-Fe, respectively, in the SrFeO_2.81_ crystal^3, 39, 40^. To obtain detailed information about the temperature dependence of the local structure around the Fe atoms, Fig. 5 presents a magnified view of feature **A** (corresponding to the NN Fe-O bond length). The figure reveals that the intensity of FT feature is minimal at 300 K for both ***E***//***ab***-plane and ***E***//***c***-axis in both warming and cooling processes. However, in Figure S2, the intensities of FT features **B** and **C** (corresponding to the NN Fe-Sr and Fe-Fe bond lengths, respectively) follow the typical thermal trend: as the temperature increases, the intensity of each FT feature decreases, suggesting the importance of Fe-O bond lengths in the local distortions of the SrFeO_2.81_ crystal. Evidently, as the temperature is increased from 40 K to 80 K, the intensity of the FT feature increases, before decreasing with a further increasing in temperature. These changes are marked in the warming and cooling cycles for the ***E***//***ab***-plane but also exist for ***E***//***c***-axis. The intensity of the FT feature is determined by the coordination number, N and the DW factor σ^2^ ^41, 42^. Figure S2(a–d) and Fig. 5(a–d) plot the EXAFS measurements in the ***ab***-plane (***E***//***ab***-plane, *θ* = 0°) and along the ***c***-axis (***E***//***c***-axis, *θ* = 70°): Normally, N does not vary with temperature, so a change in σ^2^ causes the intensity of the FT features to vary with temperature. The DW factor σ^2^ is composed of two components, σ^2^_stat_ and σ^2^(T)_vib_, which are associated with static disorder and thermal vibrations, respectively^42^. Component σ^2^_stat_ is related to the atomic structure and is unrelated to temperature, while the σ^2^(T)_vib_ is associated with the lattice vibrations, which commonly become smaller as the temperature decreases, according to the Einstein or Debye model^28, 41, 42^. As expected, at high temperature (above T_m_), reducing the temperature increases the intensity of the FT feature of the metallic/paramagnetic phase in the SrFeO_2.81_ crystal, owing to the key σ^2^(T)_vib_ factor, but below T_m_, the intensity of the FT feature decreases as the temperature declines. These anomalous results clearly reveal that σ^2^_stat_ dominates the FT intensity below T_m_, suggesting that static disorders or JT distortions that are caused by Fe 3*d* electrons have a stronger effect than the influence of temperature. The large static distortions of the octahedral/pyramidal oxygen network around the Fe ions below T_m_ can be understood as being statically contributed to static DW factors that strongly influence the FT feature of Fe-O bonds in the SrFeO_2.81_.

**Figure 6:**
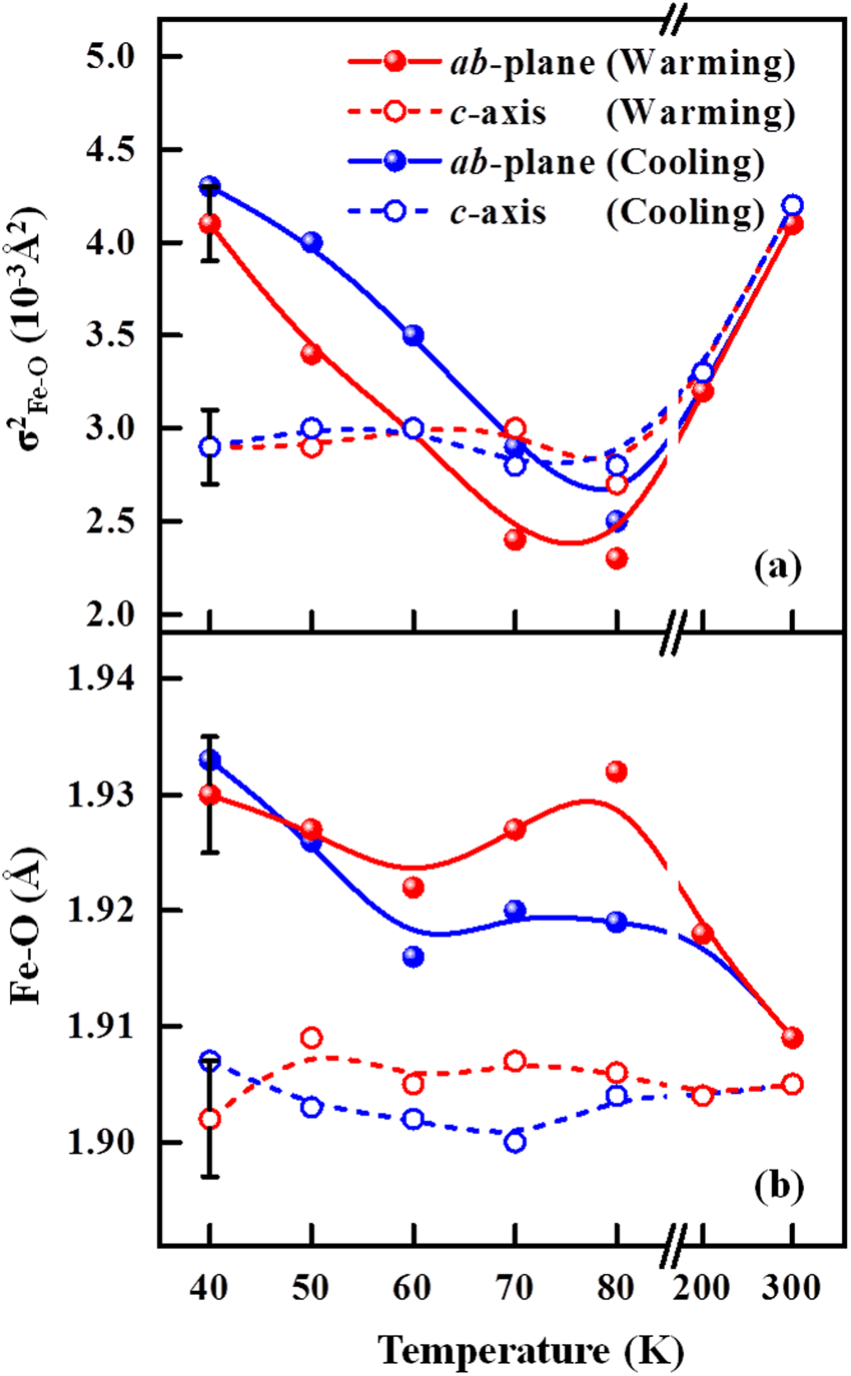
Variation of (**a**) DW factors and (**b**) NN Fe-O bond lengths with temperature, obtained by fitting temperature-dependent Fe *K*-edge EXAFS for *R* from 1.15 to 1.96 Å with angle of incidence *θ* = 0°, and *R* from 1.04 to 1.77 Å with angle of incidence *θ* = 70°.

To discuss quantitatively the local atomic structure of a single crystal of SrFeO_2.81_, fitting results for the NN Fe-O bond length, N and the DW factors are obtained using Artemis software^41, 42^ and presented in Table 1 of the Supplementary Information and Fig. 6. The DW factors and NN Fe-O bond lengths are plotted in Fig. 6 and exhibit anisotropic behavior in the ***ab***-plane and along the ***c***-axis. Generally, the DW factors that are related to atomic structure should decrease upon cooling of the sample as the thermal vibrations become less intense. However, DW factors in the ***ab***-plane abruptly increase when the temperature declines below T_m_. This sudden increase below the transition temperature [Fig. 6(a)] with thermal hysteresis indicates that the crystal structure of SrFeO_2.81_ exhibits much greater static disorder below T_m_ than above T_m_, and this phenomenon of large static disorder dominates the thermal effect. This fact can be understood with reference to soft phonon mode behavior^43, 44^, which is related to a decrease or breaking of the crystal symmetry in the ***ab***-plane of SrFeO_2.81_. Soft phonons are typically associated with phase transitions in crystals that can have more than one distinguishable lattice. The *off*-centering of the Fe ions or an order-disorder phase transition between dynamic and static distortions of Fe ions, in which interaction (that is phonon-mediated) between *off*-centered Fe and oxygen atoms drives phonon softening in the FeO_6_/FeO_5_ networks. Also, as shown in Fig. 6(a), DW factors along the ***c***-axis are nearly constant as the temperature is reduced, indicating that the thermal effect compete with static disorder along the ***c***-axis. The unusually high DW factors in the ***ab***-plane and the thermal hysteresis suggest that the local structural ordering of SrFeO_2.81_ differs between the ***ab***-plane and the ***c***-axis in both warming and cooling processes. The significant changes of DW factors in the ***ab***-plane in comparison to ***c***-axis are consistent with Reehuis *et al*.^14^. In ref. 14, using neutron diffraction studies prominent changes in the bond lengths in ***ab***-plane below transition temperature have been reported and it is anticipated to be due to orbital polarization. However, the greater value of DW factors in the ***ab***-plane during cooling than warming below T_m_ is not currently explainable. We speculate that this difference may be related to the difference between the out-of-plane and in-plane Fe 3*d* orbitals, as observed in the temperature-dependent XLD spectra in Fig. 4. Therefore, a detailed theoretical calculation may provide a quantitative description of competing lattice, orbital and spin-related degrees of freedom in this system. Figure 6(b) presents the variation of the NN Fe-O bond lengths in the ***ab***-plane and along the ***c***-axis. As evidenced from Fig. 6(b), the NN Fe-O bond lengths exhibit hysteretic behavior below the transition temperature T_m_; increase from 1.909 ± 0.005 to 1.933 ± 0.005 Å in the ***ab***-plane, and increase from 1.900 ± 0.005 to 1.909 ± 0.005 Å along ***c***-axis. Although the NN Fe-O bond lengths depend differently on temperature in the ***ab***-plane and along the ***c***-axis, the limitation on the resolution of the EXAFS analysis prevents precise determination of the Fe-O bond lengths. However, the anomalous variations of DW factors in the ***ab***-plane and along the ***c***-axis suggest that anisotropic distortion of the FeO_6_/FeO_5_ network and a change in XLD during warming and cooling processes are responsible for the anisotropy of the resistivity in the thermal hysteresis region. These results further demonstrate that the instabilities in the local Fe-O bond length/DW factors and preferred Fe 3*d e*_g_ orbitals drive the MI/metal-to-semiconductor transition in SrFeO_2.81_, in a manner similar to the driving of the Peierls MI transition in VO_2_, which was elucidated by Budai *et al*. from first-principle calculations^44^. Their calculations also revealed that increased occupation of V 3*d*_x_^2^_-y_^2^ orbitals induces the Peierls instability; lowers the total electronic energy, and opens the insulating band gap^44^. As discussed above, the XLD studies herein of the SrFeO_2.81_ crystal reveal similar phenomena. During the warming process below the transition temperature, the preferential occupation changes to in-plane Fe 3*d*_x_^2^_-y_^2^ orbitals and these changes are associated with anisotropic distortion of the FeO_6_/FeO_5_ network during both warming and cooling.

**Figure 7:**
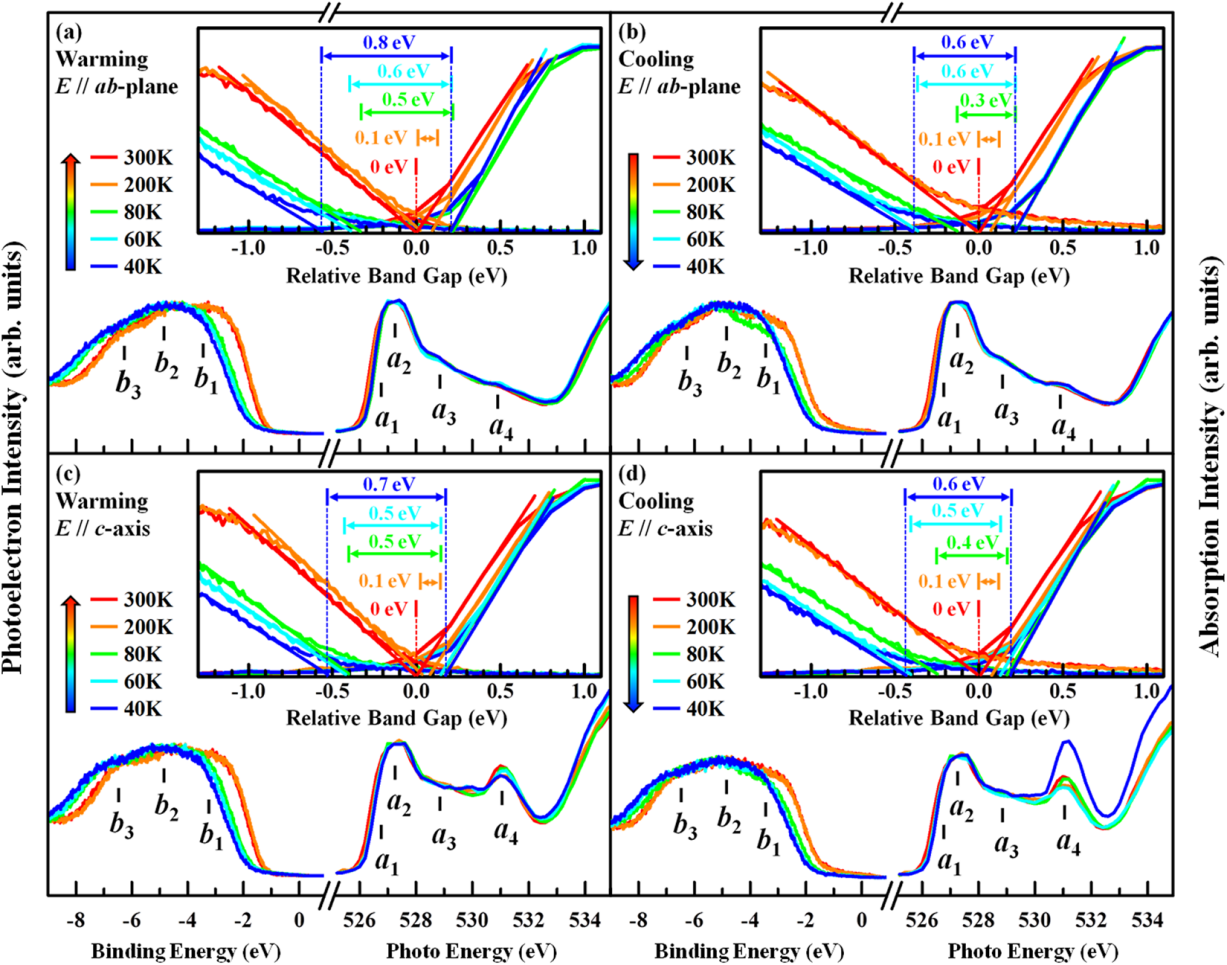
(**a**–**d**) Normalized VB-PES and O *K*-edge XANES spectra of a single crystal SrFeO_2.81_ at two angles of incidence *θ* = 0° (***E***//***ab***-plane) and 70° (***E***//***c***-axis) during warming and cooling. VB-PES spectra are obtained at photon energy of 58 eV. Insets display linear fits to VB-PES and O *K*-edge XANES spectra at various temperatures and relative band gaps.

The above assertion may also be related to possible CDW behavior in SrFeO_2.81_^45, 46^. As stated earlier, Lee *et al*.^13^ used RXS to provide evidence that satellite peaks are induced by charge modulation below the transition temperature in single crystalline tetragonal SrFeO_2.81_. As a consequence of an electron-phonon interaction, a CDW is associated with charge modulation and lattice distortion in SrFeO_2.81_^13^, as in other CDW materials^3, 19, 47^. Lattice distortion is also evident from the anomalous DW factors/bond lengths below T_m_ for the single crystal of SrFeO_2.81_, as depicted in Fig. 6. Furthermore, a CDW is a charge/electronic modulation process that arises from the coupling of valence and conduction bands, opening a band gap at/near the Fermi level (*E*_F_)^48, 49^. Therefore, to further examine the band gap opening across a MI/metal-to-semiconducting transition in SrFeO_2.81_, VB-PES spectra with photon energy hυ = 58 eV and O *K*-edge XANES spectra of SrFeO_2.81_ were obtained during warming and cooling processes, and presented in Fig. 7(a–d) for ***E***//***ab***-plane and ***E***//***c***-axis. The VB-PES spectra exhibit three major features, ***b***_**1**_, ***b***_**2**_ and ***b***_**3**_. The line shape of each feature is similar to those in the experimental spectra of SrFeO_3-δ_, LaFeO_3_ and Sr-doped LaFeO_3_^50–52^. According to the theoretical studies of SrFeO_3-δ_^50^ and Sr-doped LaFeO_3_^51–53^ at low photon energies (20 ≤ hυ ≤ 100 eV) the O 2*p* cross-section dominates the Fe 3*d* emission, so the region (from 0 to ~2 eV) at/below *E*_F_ can be attributed to the O 2*p* and Fe 3*d* (*e*_g_) hybridized states, which are essential to have a critical role in the band gap opening in SrFeO_2.81_, as discussed below. The O *K*-edge XANES spectra of SrFeO_2.81_ reflect transitions from O 1 *s* to unoccupied 2*p* states. The empty O 2*p* states are hybridized with the 3*d* and 4*sp* bands of Fe with Sr 4*d* bands^4^, so the features that are indicated by ***a***_**1**_, ***a***_**2**_, ***a***_**3**_ and ***a***_**4**_ in Fig. 7(a–d) correspond to the hybridized O 2*p*-Fe 3*d* states that are subject to crystal-field splitting, forming *t*_2g↓_, *e*_g↑_, *e*_g↓_ and *e*_g↓_ states, respectively, above/near the *E*_F_, as determined from earlier theoretical work^4^. The insets in Fig. 7(a–d) magnify VB-PES and O *K-*edge XANES spectra near *E*_F_, to elucidate the *relative band gap* opening (or energy separation) as a function of temperature. To calculate the band gap, the leading edges in both the VB-PES and O *K*-edge XANES spectra of SrFeO_2.81_ are extrapolated to the baselines to obtain valence-band maximum (*E*_VBM_) and conduction-band-minimum (*E*_CBM_)^51, 54^, respectively. At room temperature, the two extrapolated lines intersect each other, indicating the SrFeO_2.81_ sample has no band gap and exhibits metallic behavior, which is consistent with the resistivity measurement. The fact that the value of band gap at room temperature is zero in both directions, however, during both warming and cooling processes a semiconducting band gap opens up at/near the *E*_F_ and the relative value of the band gap increases as the temperature decreases. At 40 K, these values are ~0.8 and 0.6 eV for ***E***//***ab***-plane and ~0.7 and 0.6 eV for ***E***//***c***-axis, during warming and cooling processes, respectively. These results also reveal that the band gaps are almost independent of direction and whether the process in question is warming or cooling. The above results provide further evidence of an abrupt increase in resistivity at ~130 K. However, CDW modulations and the relative band gaps do not exhibit the anisotropy close to the thermal hysteresis region (~78 K), indicating that they may not depend on direction in SrFeO_2.81_ crystal or they are outside the energy resolution limit of VB-PES (~18 meV), owing to the smallness of the relevant changes. The abrupt increase in the resistivity and relative band gap opening at low temperatures provide clear evidence of the CDW nature of single crystalline SrFeO_2.81_. Since resistivity is determined by the electron-phonon coupling, it depends on the lattice distortion. We therefore believe that the sudden increase of resistivity below ~130 K in both directions is associated with the formation of the CDW, this claim is consistent with the appearance of satellite peaks in the RXS studies of Lee *et al*.^13^. However, the anisotropic behavior of resistivity in the thermal hysteresis region (~78 K) arises from anisotropic DW factors/bond lengths, as evident from the EXAFS analysis, and further stabilizes the different in-plane and out-of-plane Fe 3*d* orbitals that are observed from XLD during warming and cooling processes.

In summary, the local electronic and atomic structures of SrFeO_2.81_ were elucidated using temperature-dependent XANES and VB-PES techniques, to determine the origin of the anisotropy of resistivity in the ***ab***-plane and along the ***c***-axis in the thermal hysteresis region. The Fe *L*_3,2_-edge XLD results reveal that, during cooling from room temperature to below T_m_, the Fe 3*d* electrons preferentially occupy the out-of-plane Fe 3*d*_3z_^2^_-r_^2^ orbitals. However, during warming below T_m_, they preferentially occupy the in-plane Fe 3*d*_x_^2^_-y_^2^ orbitals. Local atomic structural analysis of the temperature-dependent Fe *K*-edge EXAFS of SrFeO_2.81_ reveals unusually large DW factors with thermal hysteresis below T_m_. Additionally, NN Fe-O bond lengths exhibit anisotropy in the ***ab***-plane. Experimental results suggest that the local atomic structural ordering in the ***ab***-plane differs from that along the ***c***-axis during both warming and cooling processes. This distinction stabilizes the difference between the forms of atomic structural ordering in the warming and cooling processes and is responsible for the anisotropy of resistivity below T_m_ in the thermal hysteresis region in the ***ab***-plane and along the ***c***-axis in a single crystal of SrFeO_2.81_. The abrupt increase in resistivity and evidence of relative band gap opening that were obtained from the O *K*-edge XANES and VB-PES experiment, along with the presence of satellite peaks in RXS spectra^13^, confirm the CDW nature of the SrFeO_2.81_ single crystal at low temperatures.

## Methods

### Sample preparation and characterization

High-quality single crystals of SrFeO_2.81_ were prepared using the floating zone method^27^. In ref. 13 reported by Lee *et al*., the estimated number of oxygen-content (2.875) in SrFeO_x_ is based from their XRD measurements, however, they did not employ techniques such as thermogravimetric analysis of iodometric titration to determine the oxygen content. Meanwhile, the refined parameter of XRD cannot tell structure difference between SrFeO_2.81_ and SrFeO_2.875_ because of the insensitivity of X-ray to oxygen atom. Further, according to the refs 1 and 2, and introduction section of the present work, SrFeO_x_ with oxygen-content x = 2.875 is reported to be pure tetragonal, however for x = 2.75, it is pure orthorhombic, so our sample (SrFeO_2.81_) with oxygen-content x = 2.81 lies in between these two and also XRD presented in Fig. 1 confirm the tetragonal nature of the sample. The magnetic susceptibility and electrical resistivity were measured using a superconducting quantum interference device and a physical property measurement system, respectively. XRPD patterns were obtained on an image plate at beamline-01C of the National Synchrotron Radiation Research Center (NSRRC) in Hsinchu, Taiwan, using X-ray with a wavelength of 0.728 Å (16 keV). Lebail refinements of XRPD patterns were performed using the Fullprof software package^55–57^. Fe *L*_3,2_-edge XANES/XLD, *K*-edge XANES/EXAFS, O *K*-edge XANES and VB-PES (incident photon energy, hυ = 58 eV) experiments were conducted at the Dragon-11A, Wiggler-17C and U9-ARPES BL21B1 beamlines at the NSRRC. The measurements were made during warming and cooling at various temperatures to elucidate the local electronic and atomic structures of the SrFeO_2.81_ single crystal. Further, in the warming and cooling process, all the experiments have been carried out in the same conditions. In the warming process, sample is heated from the lowest temperature (30 K) to 40 K. At 40 K, sample temperature has been kept constant for ~30 mins and then data have been collected for two orientations of sample. This is repeated for all temperatures and during cooling process too. The temperature of the sample was controlled using a closed-cycle refrigerator with an accuracy of ±0.1 K. Local atomic structure analysis of EXAFS data was performed using the Artemis program. Artemis combines the multiple-scattering EXAFS computer program FEFF^41^ and the nonlinear least-squares-fitting computer program FEFFIT^42^.

## Electronic supplementary material

Supplementary Information
