# Supplementary material for: Anisotropy in the thermal hysteresis of resistivity and charge density wave nature of single crystal SrFeO3-δ: X-ray absorption and photoemission studies
Source: Sci Rep. 2017 Mar 13;7:161. doi: 10.1038/s41598-017-00247-z (PMC5428035; doi:10.1038/s41598-017-00247-z)
Supplement: Supplementary file 1 — Supplementary Information [file 41598_2017_247_MOESM1_ESM.doc]

**Supplementary Information**

**Anisotropy in the thermal hysteresis of resistivity and charge density wave nature of single crystal SrFeO_3-δ_: X-ray absorption and photoemission studies**

S. H. Hsieh,^1, §^ R. S. Solanki,^1, §, ϯ^ Y. F. Wang,^1^ Y. C. Shao,^1^ S. H. Lee,^1^ C. H. Yao,^1^ C. H. Du,^1^ H. T. Wang,^2^ J. W. Chiou,^3^ Y. Y. Chin,^4^ H. M. Tsai,^4^ J.-L. Chen,^4^ C.W. Pao,^4^ C.-M. Cheng,^4^ W.-C. Chen,^4^ H. J. Lin,^4^ J. F. Lee,^4^ F. C. Chou,^5^ W. F. Pong^1,*^

^1^ Department of Physics, Tamkang University, Tamsui 251, Taiwan

^2^ Department of Physics, National Tsinghua University, Hsinchu 300, Taiwan

^3^ Department of Applied Physics, National University of Kaohsiung, Kaohsiung 811, Taiwan

^4^ National Synchrotron Radiation Research Center, Hsinchu 300, Taiwan

^5^ Center for Condensed Matter Sciences, National Taiwan University, Taipei 106, Taiwan


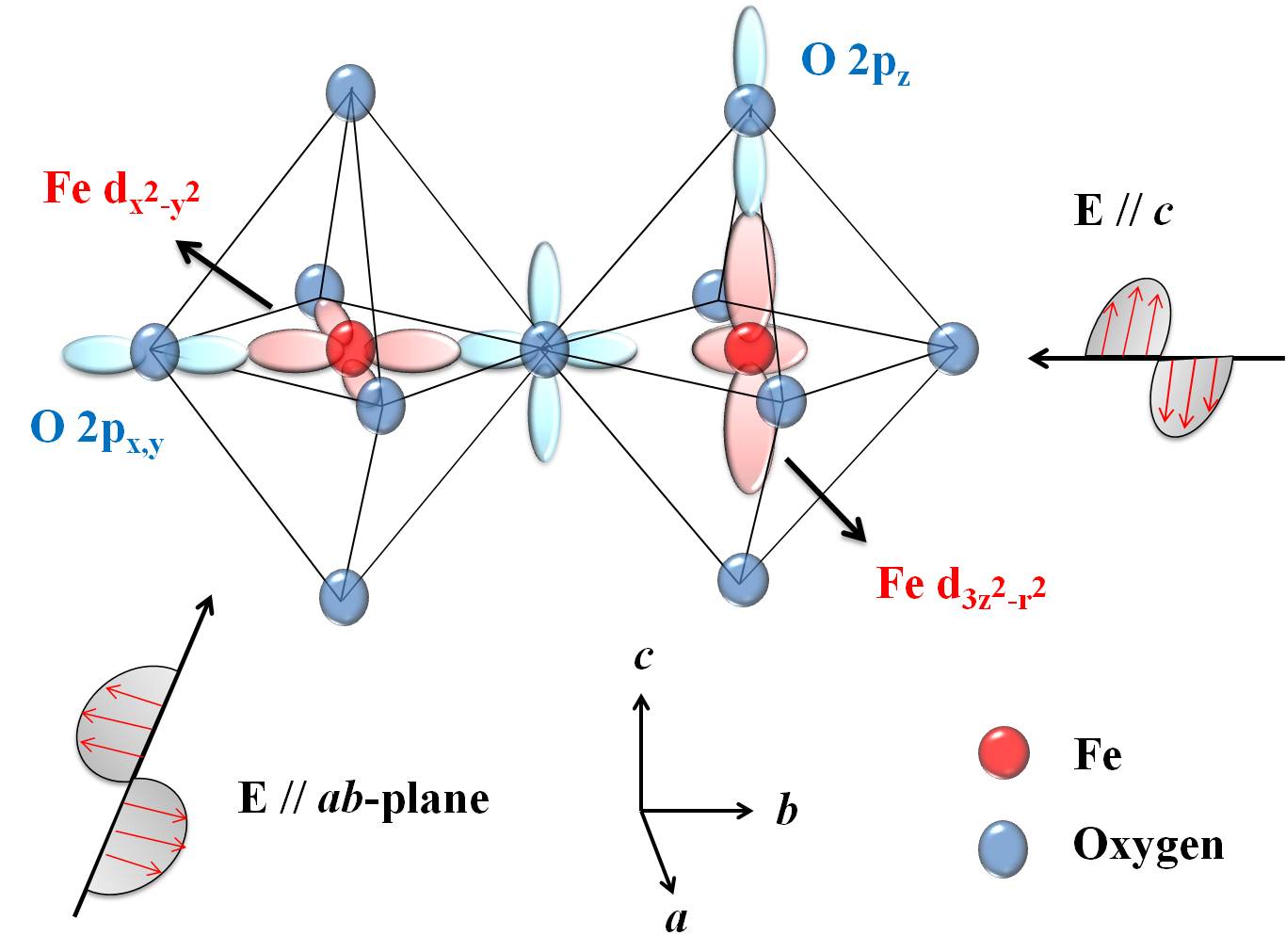
**Figure S1:** Schematic representation to elucidate out-of-plane and in-plane 3*d* states. The direct hybridization of Fe 3*d*_x_^2^_-y_^2^-O 2*p*_x, y_ (in-plane) and Fe 3*d*_3z_^2^_-r_^2^-O 2*p*_z_ (out-of-plane) are probed by electric field ***E*** parallel to the ***ab***-plane (angle of incidence, *θ*= 0) and electric field ***E*** nearly parallel to the ***c***-axis (angle of incidence, *θ*= 70), respectively.

**Figure S2:** The magnitude of the FT spectra of the temperature dependent Fe *K*-edge EXAFS **(a)** and **(b)**: in a *k* range from 2.65 to 11.51 Å^-1^ at angle of incidence *θ*= 0^o^ (***E***//***ab***-plane); **(c)** and **(d)**: in a *k* range and from 2.65 to 11.51 Å^-1^ at angle of incidence *θ*= 70^o^ (***E***//***c***-axis) on warming and cooling process. Three main FT features **A**, **B** and **C**, which correspond to the nearest-neighbor Fe-O, Fe-Sr and Fe-Fe bond distances for SrFeO_2.81_ are marked with vertical lines. Insets show the *k*^2^χ dependence as a function of *k*.

**
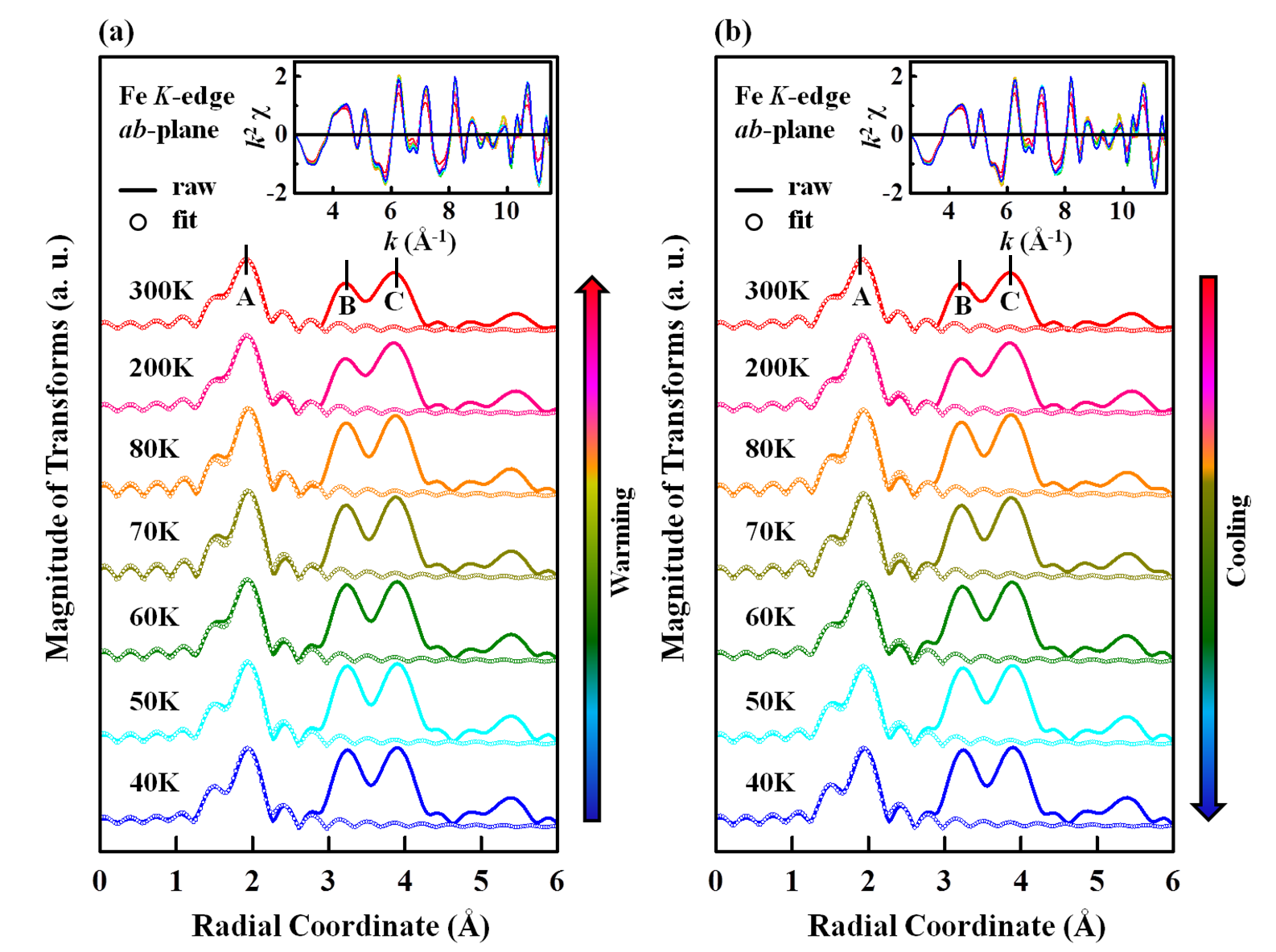
**

**
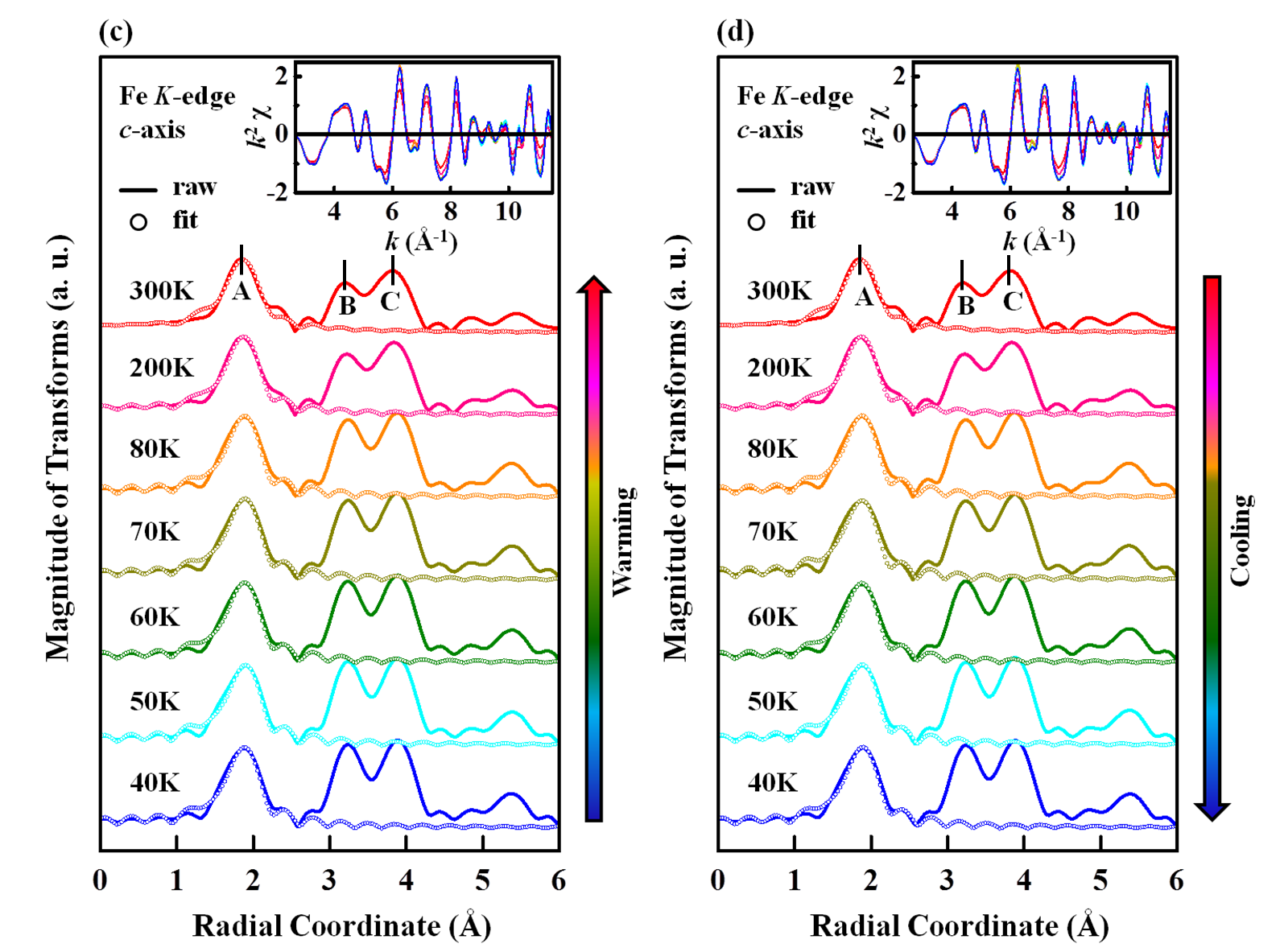
**

**Table 1:** Best fit parameters obtained from the fitting of Fe *K*-edge EXAFS data in the *R*-space mode from 1.15 to 1.96 Å for angle of incidence *θ*= 0^o^ (***E***//***ab***-plane) and from 1.04 to 1.77 Å for angle of incidence *θ*= 70^o^ (***E***//***c***-axis) during warming and cooling process. N_ab_, σ^2^_ab_, and *R*_ab_ & N_c_, σ^2^_ab_, and *R*_c_ correspond to number of nearest neighbor (NN) oxygen ions around central Fe ion, square of Debye-Waller factor for Fe-O, and average Fe-O bond length in the ***ab***-plane and ***c***-axis, respectively. In the pyramidal coordination along ***c***-axis there is only one NN oxygen for Fe, however in the octahedral they are two, therefore the average value of NN along ***c***-axis (N_c_) has been around 1.5± 0.1, for the ***ab***-plane as usual the NN oxygen atoms (N_ab_) for both pyramidal and octahedral coordination are around 4.0± 0.1.

| Temperature | N_ab_ | N_c_ | σ_ab_^2^  (×10^-3^Å^2^) | σ_c_^2^  (×10^-3^Å^2^) | R_ab_ (Å) | R_c_ (Å) |
| --- | --- | --- | --- | --- | --- | --- |
| **Cooling** |  |  |  |  |  |  |
| 300K | 4± 0.1 | 1.5± 0.1 | 4.1 ± 0.2 | 4.2 ± 0.2 | 1.909 ± 0.005 | 1.905 ± 0.005 |
| 200K | 4± 0.1 | 1.5± 0.1 | 3.2 ± 0.2 | 3.3 ± 0.2 | 1.918 ± 0.005 | 1.904 ± 0.005 |
| 80K | 4± 0.1 | 1.5± 0.1 | 2.5 ± 0.2 | 2.7 ± 0.2 | 1.919 ± 0.005 | 1.904 ± 0.005 |
| 70K | 4± 0.1 | 1.5± 0.1 | 2.9 ± 0.2 | 3.0 ± 0.2 | 1.920 ± 0.005 | 1.900 ± 0.005 |
| 60K | 4± 0.1 | 1.5± 0.1 | 3.5 ± 0.2 | 3.0 ± 0.2 | 1.916 ± 0.005 | 1.902 ± 0.005 |
| 50K | 4± 0.1 | 1.5± 0.1 | 4.0 ± 0.2 | 2.9 ± 0.2 | 1.926 ± 0.005 | 1.903 ± 0.005 |
| 40K | 4± 0.1 | 1.5± 0.1 | 4.3 ± 0.2 | 2.9 ± 0.2 | 1.933 ± 0.005 | 1.907 ± 0.005 |
| **Warming** |  |  |  |  |  |  |
| 40K | 4± 0.1 | 1.5± 0.1 | 4.1 ± 0.2 | 2.9 ± 0.2 | 1.930 ± 0.005 | 1.902 ± 0.005 |
| 50K | 4± 0.1 | 1.5± 0.1 | 3.4 ± 0.2 | 3.0 ± 0.2 | 1.927 ± 0.005 | 1.909 ± 0.005 |
| 60K | 4± 0.1 | 1.5± 0.1 | 3.0 ± 0.2 | 3.0 ± 0.2 | 1.922 ± 0.005 | 1.905 ± 0.005 |
| 70K | 4± 0.1 | 1.5± 0.1 | 2.4 ± 0.2 | 2.8 ± 0.2 | 1.927 ± 0.005 | 1.907 ± 0.005 |
| 80K | 4± 0.1 | 1.5± 0.1 | 2.3 ± 0.2 | 2.8 ± 0.2 | 1.932 ± 0.005 | 1.906 ± 0.005 |
| 200K | 4± 0.1 | 1.5± 0.1 | 3.2 ± 0.2 | 3.3 ± 0.2 | 1.918 ± 0.005 | 1.904 ± 0.005 |
| 300K | 4± 0.1 | 1.5± 0.1 | 4.1 ± 0.2 | 4.2 ± 0.2 | 1.909 ± 0.005 | 1.905 ± 0.005 |
